# Supplementary material for: Interpreting SNP heritability in admixed populations
Source: Genetics. 2025 May 22;230(4):iyaf100. doi: 10.1093/genetics/iyaf100 (PMC12273224; doi:10.1093/genetics/iyaf100)
Supplement: iyaf100_Supplementary_Data [file iyaf100_supplementary_data.zip › Supplemental_Material_GENETICS-2025-308022.pdf]

Supplement

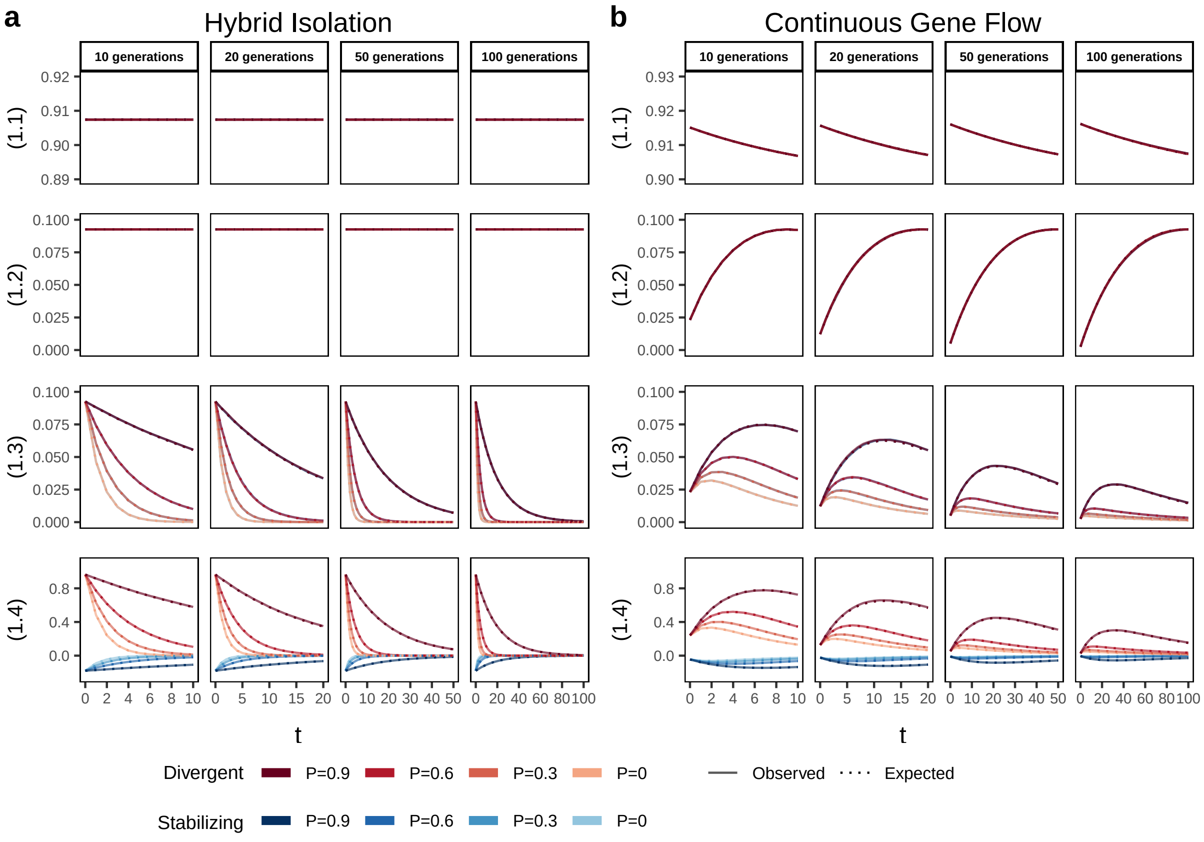

Figure S1 The behavior of the four components of genetic variance in admixed populations under the (A) HI and (B) CGF models. We assume that the mean ancestry proportion in the population is 0.5. The solid lines represent values observed in simulations averaged across ten replicates and the dotted lines represent the expected values based on Eq. 1 of the main text. The red and blue lines represent values for traits 1 and 2, respectively. P indicates the strength of assortative mating. P=0.6 is missing for simulations run for 50 and 100 generations and $\theta \in \{0.1, 0.2\}$ due to the difficulty in finding mate pairs (Methods).

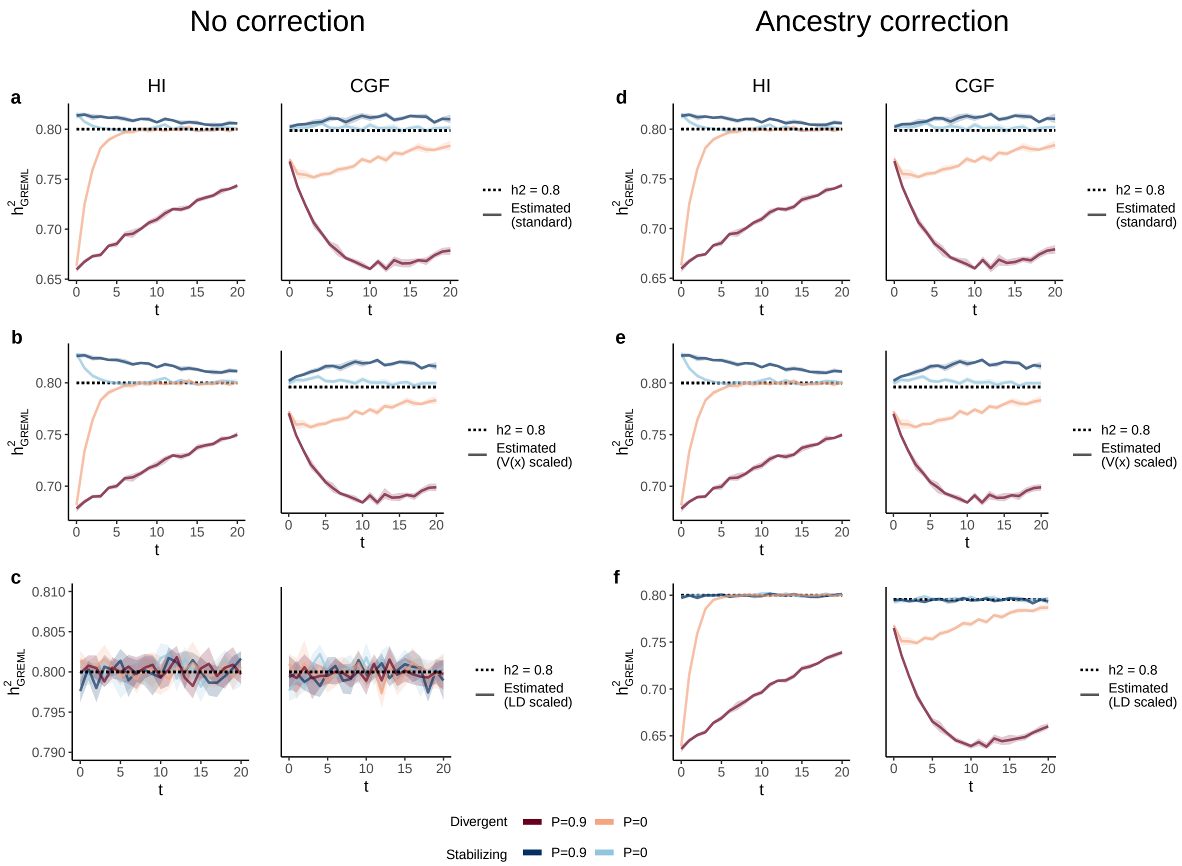

Figure S2 The behavior of GREML estimates of SNP heritability ($\hat{h}^2_{snp}$) in admixed populations under the HI (left column) and CGF (right column) models either without (A-C) or with (D-F) individual ancestry as a fixed effect. The solid lines represent $\hat{h}^2_{snp}$ averaged across ten replicates, with red and blue colors representing estimates for traits under divergent and stabilizing selection, respectively. (A, D) show the behavior of $\hat{h}^2_{snp}$ for the default scaling, (B, E) shows $\hat{h}^2_{snp}$ when the genotype at a locus is scaled by its sample variance ($\mathbb{V}(x)$ scaled), and (C, F) when it is scaled by the sample covariance (LD scaled). The shaded area represents the 95% confidence bands generated by bootstrapping (sampling with replacement 100 times) the point estimate reported by GCTA. The black dotted lines represent the expected heritability value given the simulation settings ($h^2 = 0.8$). P indicates the strength of assortative mating

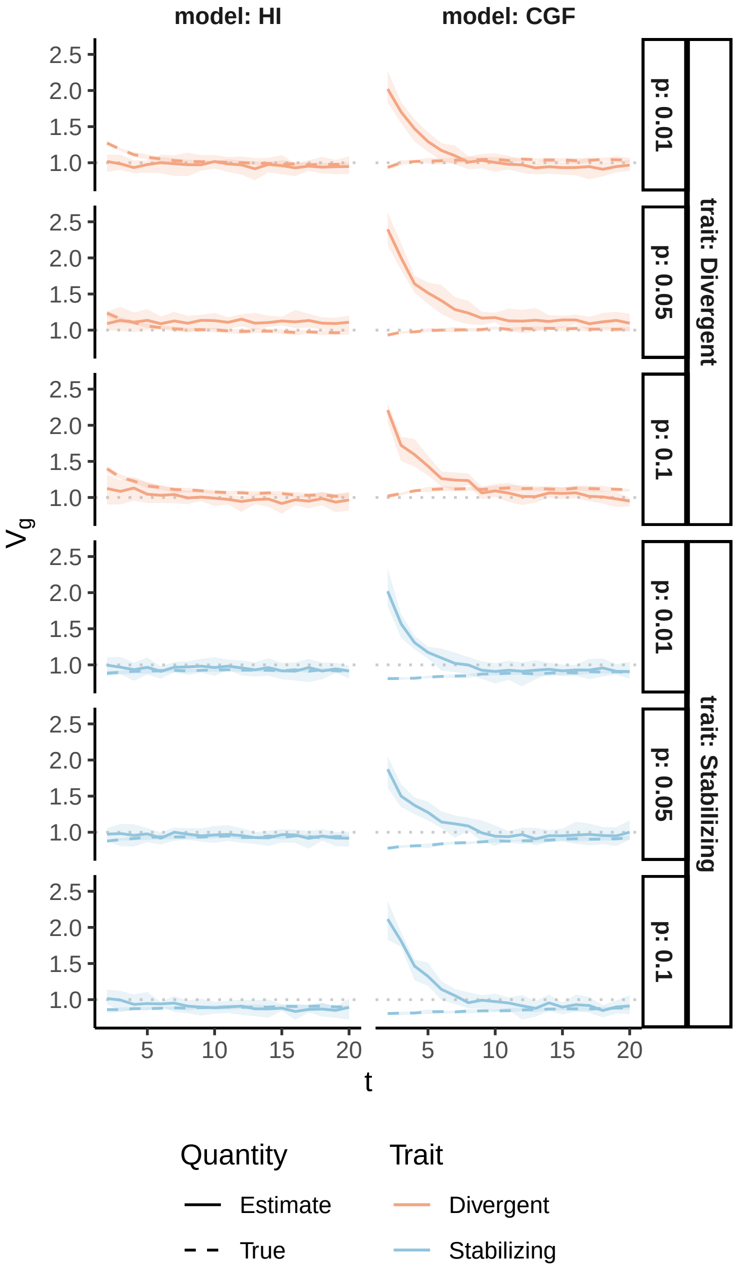

Figure S3 Behavior of LDSC estimates of V_g under the HI and CGF admixture models as a function of time since admixture (x-axis) for traits under directional (blue) and stabilizing selection (red) and with $p \in \{0.01, 0.05, 0.1\}$ proportion of causal variants. The dashed lines indicate (A) the simulated V_g whereas the solid lines show the estimated value. Dotted horizontal lines indicate the expected genic variance. Shaded ribbons indicate the 95% CI across ten replicates.

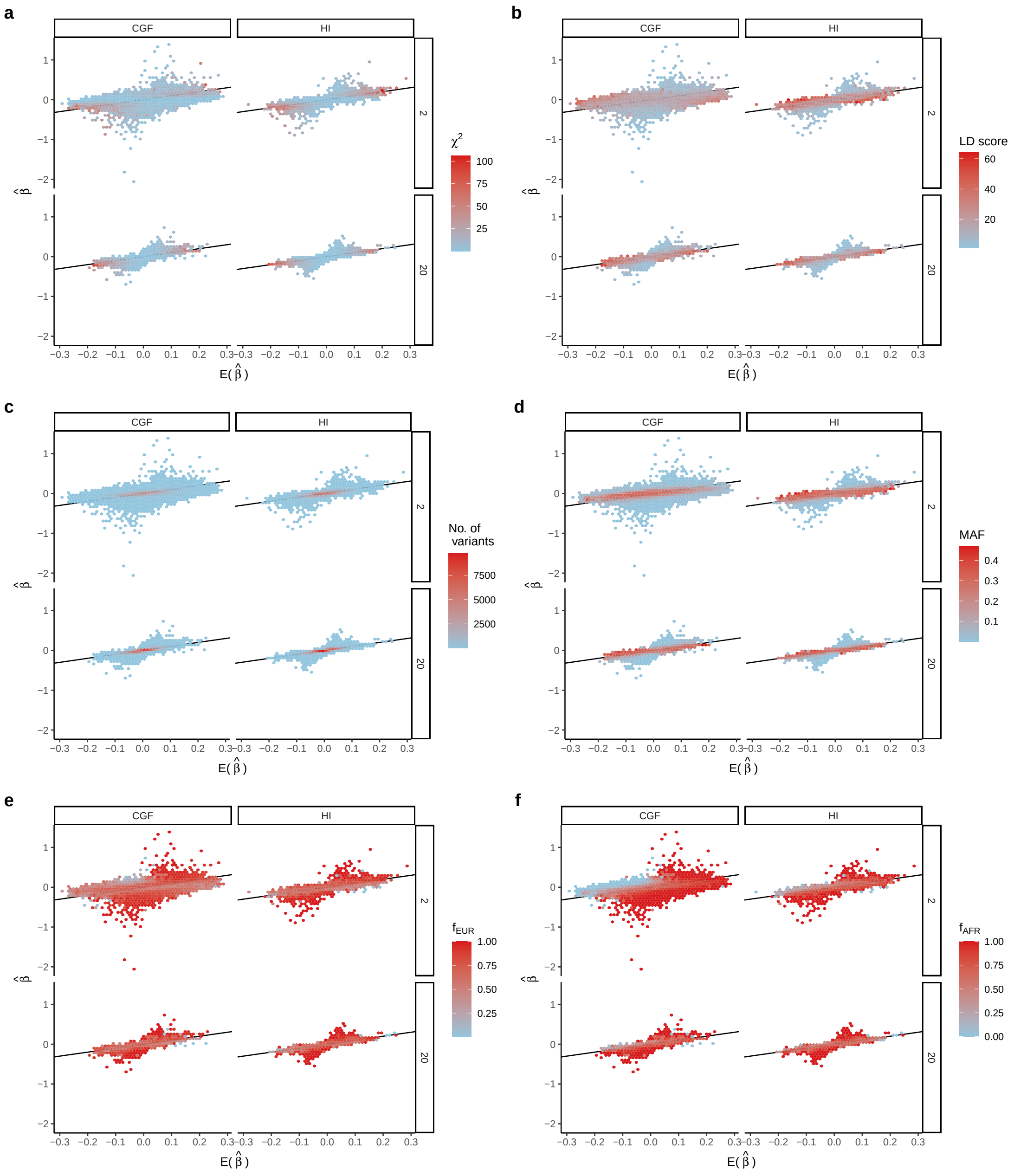

Figure S4 Expected vs Observed effect sizes of 87,564 variants on chromosome 2 for a GWAS carried out in 5,000 admixed individuals simulated under the HI and CGF models (panel columns) for generations 2 and 20 (panel rows) since admixture. Variants are grouped into bins and each bin is colored by the (A) mean χ^2, (B) mean LD score, (C) no. of variants, (D) mean MAF, (E) mean CEU frequency, and (F) mean YRI frequency across variants in that bin. The $\mathbb{E}(\hat{\beta}_j)$ of the jth SNP was computed as $\sum_{i=1}^{m} r_{ij}\beta_i$ where β_i is the simulated effect of the ith causal variant and r_{ij} is the genotypic correlation between them. The plot shows overdispersion in $\hat{\beta}$ due to population structure, especially in the 2nd generation of the CGF model. This appears to be driven by incoming variants that are common in CEU (E) but rare or absent in YRI (F).

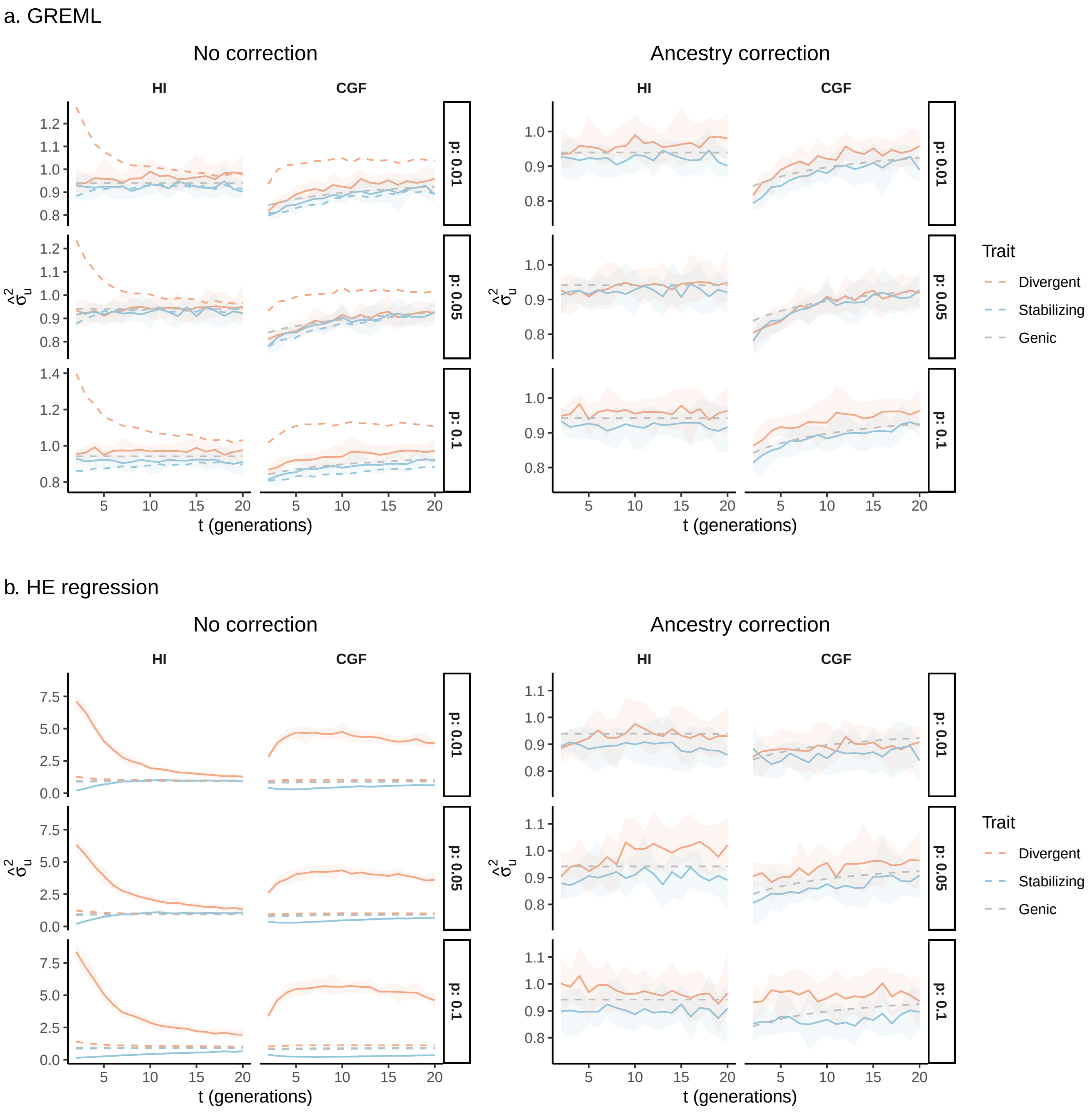

Figure S5 (A) GREML and (B) HE estimates of V_g (y-axis) on simulated genotype data of 5,000 admixed individuals for chromosome 2 under the HI and CGF models as a function of time since admixture (x-axis) and trait architecture (color). Results are also shown for varying proportion of causal variants (p) and whether or not ancestry (20 PCs) were included as covariates in the model. GREML and HE estimates are consistent with analytical expectations and simulations of unlinked markers.

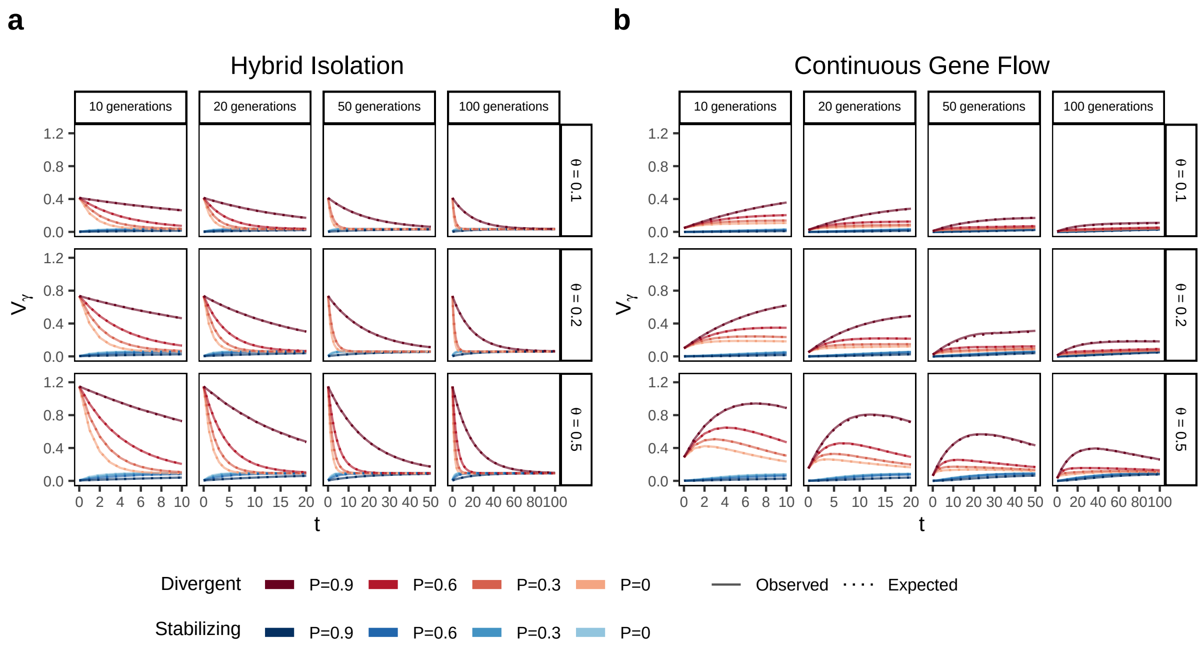

Figure S6 The behavior of the genetic variance due to local ancestry in admixed populations under the (A) HI and (B) CGF models. The solid lines represent values observed in simulations averaged across ten replicates and the dotted lines represent the expected values based on Eq. 1 of the main text. The red and blue lines represent values for traits 1 and 2, respectively. P indicates the strength of assortative mating. P=0.6 is missing for simulations run for 50 and 100 generations and $\theta \in \{0.1, 0.2\}$ due to the difficulty in finding mate pairs (Methods).

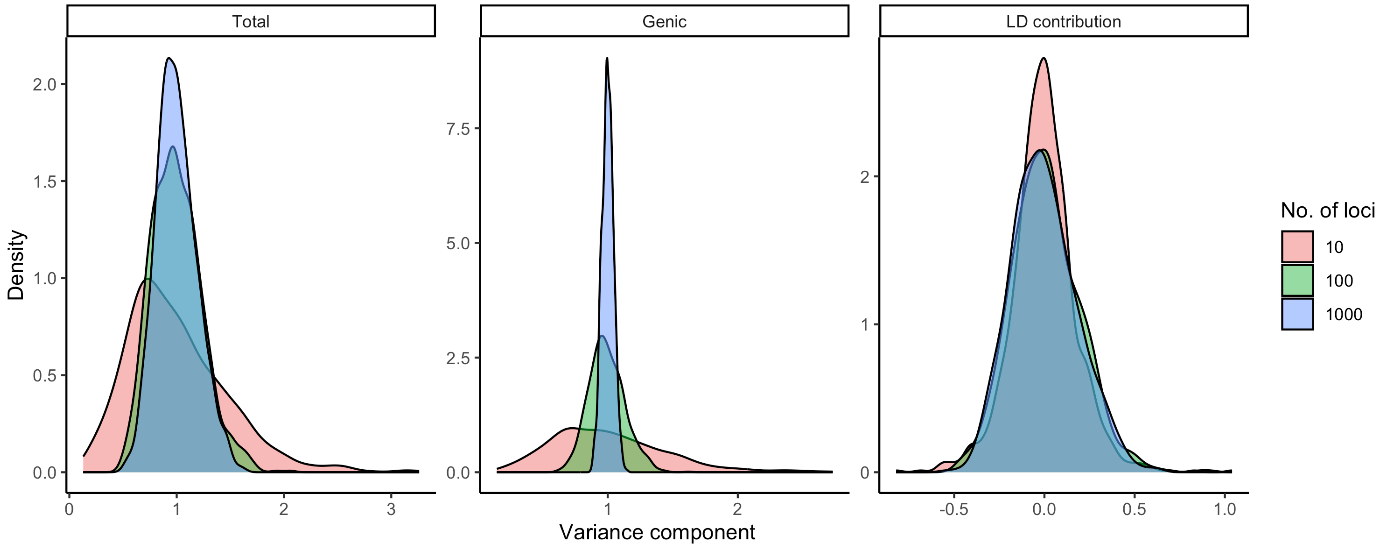

Figure S7 Distribution of the total genetic variance (left), genic variance (middle), and LD component (right) for a neutral trait simulated by drawing effects for 10, 100, or 1,000 causal variants in ASW. The total genetic variance is the sum of the genic and LD components.

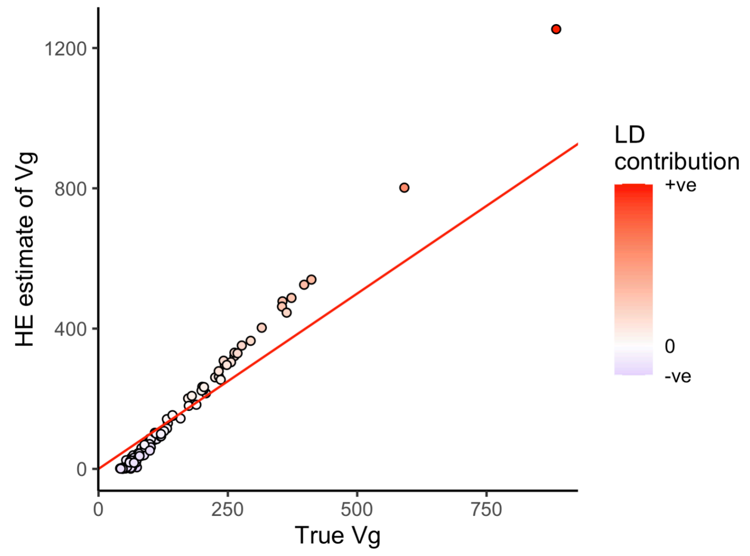

Figure S8 The effect of directional LD on Haseman-Elston estimate of genetic variance (V_g). Each individual point is an independent simulation where the effects were drawn from a normal distribution and applied to genotypes from an admixed population (Methods). The solid red line shows the $y = x$ line and the color of each point represents the contribution of LD to V_g.

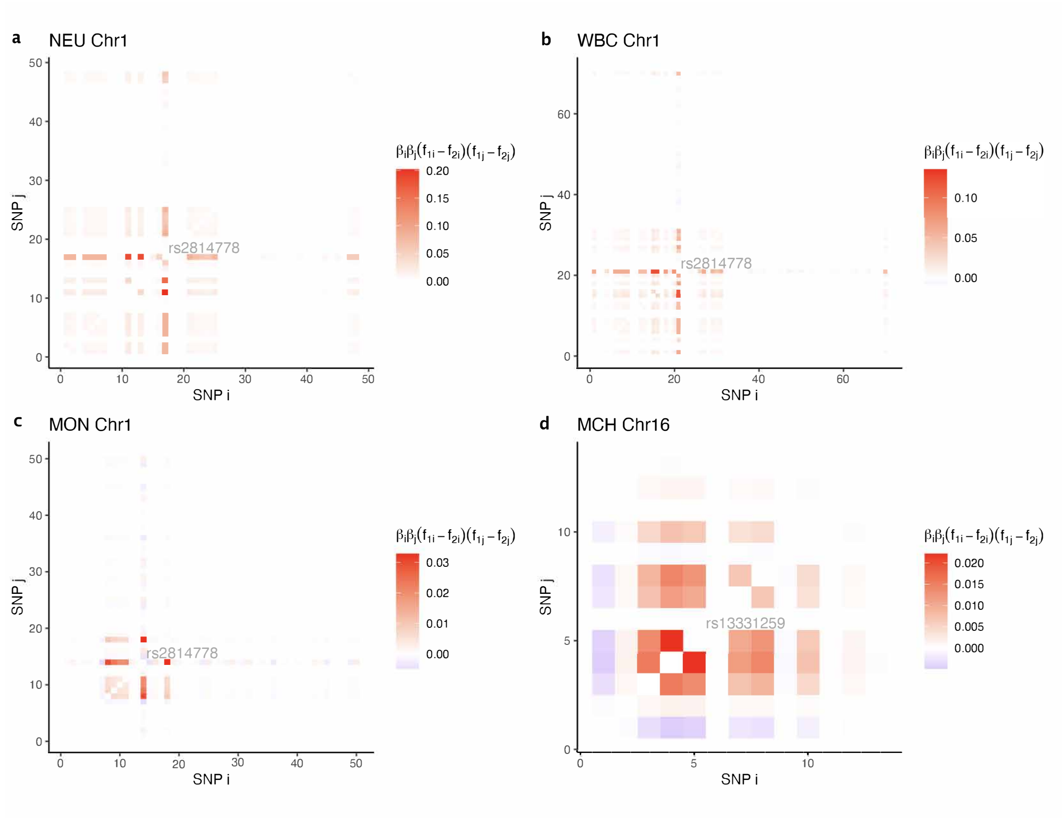

Figure S9 The LD contribution to the variance explained by variant pairs for (A) neutrophil counts (NEU), (B) white blood count (WBC), (C) monocyte count (MON), and (D) mean corpuscular hemoglobin (MCH). Only chromosomes where we suspected there was a disproportionate contribution to the variance explained are shown.

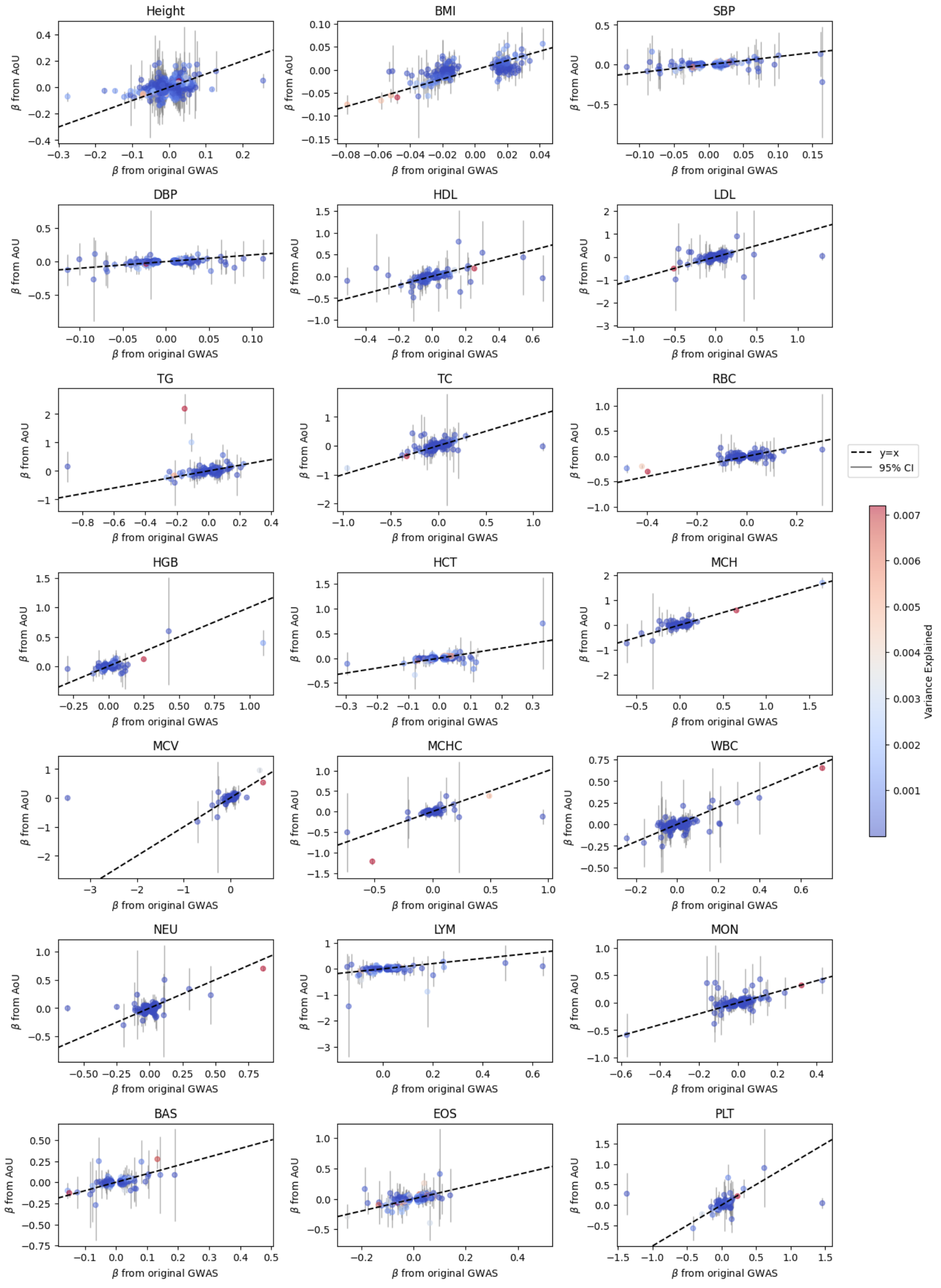

Figure S10 Effect sizes estimated in the AoU cohort compared to effect sizes from original GWAS summary statistics (Methods). Each point is a variant colored by the amount of variance it explains. Variance explained is computed as $\hat{\beta}^2 2f(1-f)$, where f is the minor allele frequency in the African American AoU cohort. The $y = x$ line is shown as a dotted black line and the gray lines are 95% confidence intervals of $\hat{\beta}$ in the AoU cohort.
